# Donor γδT Cells Promote GVL Effect and Mitigate aGVHD in Allogeneic Hematopoietic Stem Cell Transplantation

**DOI:** 10.3389/fimmu.2020.558143

**Published:** 2020-10-16

**Authors:** Yuan Song, Ying Zhu, Bo Hu, Yonghao Liu, Dandan Lin, Ziqi Jin, Zhinan Yin, Chen Dong, Depei Wu, Haiyan Liu

**Affiliations:** ^1^Immunology Programme, Life Sciences Institute and Department of Microbiology and Immunology, Yong Loo Lin School of Medicine, National University of Singapore, Singapore, Singapore; ^2^Institute of Blood and Marrow Transplantation, Jiangsu Institute of Hematology, The First Affiliated Hospital of Soochow University, Collaborative Innovation Center of Hematology, National Clinical Research Center for Hematologic Diseases, Soochow University, Suzhou, China; ^3^Zhuhai Precision Medical Center, Zhuhai People’s Hospital (Zhuhai Hospital Affiliated With Jinan University), Jinan University, Zhuhai, China; ^4^The Biomedical Translational Research Institute, Faculty of Medical Science, Jinan University, Guangzhou, China; ^5^Institute for Immunology and School of Medicine, Tsinghua University, Beijing, China

**Keywords:** γδT cells, IL-17, graft-versus-host disease, graft-versus-leukemia, hematopoietic stem cell transplantation

## Abstract

Disease relapse and graft-versus-host disease (GVHD) are the major complications affecting the outcomes of allogeneic hematopoietic stem cell transplantation (allo-HSCT). While the functions of αβT cells are extensively studied, the role of donor γδT cells in allo-HSCT is less well defined. Using TCRδ^-/-^ donors lacking γδT cells, we demonstrated that donor γδT cells were critical in mediating graft-versus-leukemia (GVL) effect during allo-HSCT. In the absence of donor γδT cells, IFN-γ production by CD8^+^ T cells was severely impaired. Vγ4 subset was the major γδT cell subset mediating the GVL effect *in vivo*, which was partially dependent on IL-17A. Meanwhile, donor γδT cells could mitigate acute GVHD in a murine allo-HSCT model by suppressing CD4^+^ T cell activation and the major γδT cell subset that exerted this protective function was also Vγ4 γδT cells. Therefore, our findings provide evidence that donor γδT cells, especially Vγ4 subset, can enhance GVL effect and mitigate aGVHD during allo-HSCT.

## Introduction

Allogeneic hematopoietic stem cell transplantation (allo-HSCT) is one of the most curative options for treating leukemia and other hematopoietic malignant diseases (1, 2). But its efficacy is limited by graft-versus-host disease (GVHD) and disease relapse (3). GVHD is induced by an immune response of donor T cells against recipient healthy tissues (4). T cells are comprised of two major subpopulations, identified by their expression of either αβ or γδ TCR heterodimers. Donor αβT cells are thought to be the primary T cell subpopulation responsible for mediating GVHD and graft versus leukemia (GVL) responses during allo-HSCT (5, 6). Nevertheless, a number of recent studies suggest that γδT cells might also play a critical role in mediating the outcomes of allo-HSCT (1, 7, 8).

γδT cells are present in relatively smaller numbers and percentages in most tissues of mouse and human compared to αβT cells (9). Generally, only a small portion of γδT cells express CD4 or CD8 co-receptors. They can be activated by stress-induced ligands without the antigen presentation *via* major histocompatibility complex (MHC). The ligands of γδTCR include MHC-related and MHC-unrelated molecules. It is not clear which endogenous ligands activate γδT cells in most disease conditions. γδT cells also exhibit similar recognition mechanisms as NK cells. They can express NKG2D and KIRs, and recognize target cells expressing stress-induced ligands (10). Binding of ligands to activating receptors on γδT cells triggers cytotoxicity by releasing cytotoxic granules and induces immune regulatory functions by producing cytokines (11).

Previous studies demonstrated that γδT cells might facilitate allogeneic engraftment and contribute to anti-viral immunity (12). A recent study showed that human γδT cells were quickly reconstituted with radically altered but stable TCR repertoires after HSCT (13). In this study, they also observed a few individual γδT cell clones (mainly but not exclusively within the Vγ9 and Vδ2 fraction) underwent additional massive proliferation in response to cytomegalovirus (CMV). In another study, the T cell receptor gamma (TRG) repertoire of γδT cells within peripheral blood stem cells was analyzed by using next-generation sequencing technology. The results showed that the grafts from CMV^+^ donors presented a reshaped TRG repertoire, and the TRG composition was not associated with aGVHD development (14). It has been reported that Vδ2^-^ γδT cells were significantly expanded in CMV-seropositive transplant recipients and these cells can directly lyse CMV-infected cells (15). Adoptive transfer of human Vγ9Vδ2 T cells expanded with phosphorylated antigens could effectively prevent the progress of Epstein-Barr virus-induced lymphoproliferative disease in humanized mice (16). These studies explored the γδT cell responses in anti-viral immunity and the potential of using adoptive γδT cell immunotherapy in allogeneic transplantation recipients.

γδT cells can mediate innate anti-tumor activity by direct cytotoxicity and IFN-γ production (17). However, γδT cells have also been reported to promote tumor growth by producing IL-17 (18, 19). Many studies in clinical trials have demonstrated the anti-leukemia effect of human γδT cells in haematological malignancies after allo-HSCT. An eight years’ follow-up study indicated a survival advantage in patients with increased γδT cells after allo-HSCT (20). AML and ALL patients recovered with high γδT cell numbers displayed a better leukemia-free survival (LFS) and overall survival (OS) compared with those with low γδT cell numbers. Interestingly, there was no increase in the incidence of acute GVHD (aGVHD) associated with high γδT cell numbers. Moreover, human γδT cells from blood of patients showed significant cytotoxicity against multiple myeloma or lymphoma cells (21–23). Treatment of paediatric ALL patients with zoledronate was associated with an increase of Vδ2 γδT cells and an increase of the cytotoxicity against primary leukemia blasts (24). Although the anti-tumor function of γδT cells has been suggested by many studies, it is still not clear which γδT subset possesses a strong anti-tumor effect and whether this effect is also mediated through regulation of αβT cells besides direct cytotoxicity after allo-HSCT.

There is evidence suggesting that γδT cells are not the primary initiators of GVHD (25). Although an increased number of γδT cells were found in patients who developed aGVHD up to three months after allo-HSCT (26), a subsequent study found no significant correlation between γδT cell recovery and the incidence of GVHD in the first 12 months post HSCT (27). In fact, a recent study showed improved OS, LFS, and less GVHD in patients with high immune reconstitution of γδT cells two months after allo-HSCT (8). In murine studies, donor γδT cells have been shown to exacerbate aGVHD and the elimination of γδT cells from donor mice significantly reduced the lethality of GVHD (28). Similarly, another study showed that co-infusion of *in vitro* expanded donor-derived γδT cells and naïve αβT cells on the same day post allo-HSCT significantly exacerbated GVHD (29). However, donor-derived γδT cell infusion resulted in reduced GVHD and improved survival when the administration of naïve αβT cells was delayed for 2 weeks. This protective effect of γδT cells is mediated indirectly *via* donor BM-derived αβT cells. Therefore, donor-derived γδT cells could exert anti-leukemia effect while protecting the host from GVHD. However, this notion has not been fully examined in animal models and the detailed mechanism is not known.

In this study, by performing allo-HSCT using TCRδ^-/-^ donors and γδT cell infusions, we investigated the role of donor γδT cells in both GVL and aGVHD murine models. Our results suggest that donor Vγ4 γδT cells could promote GVL and suppress aGVHD in allo-HSCT through the regulation of αβT cell immune responses.

## Materials and Methods

### Mice

Specific pathogen free C57BL/6 (H2K^b^) and BALB/c (H2K^d^) mice (aged 6–8 weeks) were purchased from Shanghai Laboratory Animal Center (Shanghai, China) and In Vivos (Singapore). CD45.1-C57BL/6 (H2K^b^) mice were obtained from Beijing Vital River Laboratory Animal Technology Co. Ltd (Beijing, China) and In Vivos (Singapore). TCR-δ^-/–^C57BL/6 (H2K^b^) mice were provided by Prof. Zhinan Yin (Jinan University, Guangzhou, China). TCR-β^-/–^C57BL/6 (H2K^b^) mice were purchased from The Jackson Laboratory (Sacramento, CA). TCR-β^-/–^CD45.1-C57BL/6 (H2K^b^) mice were generated by crossing CD45.1 with TCR-β^-/-^. IL-17A^−/−^-C57BL/6 mice were provided by Dr. Chen Dong (Tsinghua University, Beijing, China). All mice are female and maintained in specific pathogen-free conditions and in accordance with the guidelines approved by the Institutional Laboratory Animal Care and Use Committee of Soochow University and National University of Singapore.

### Cell Lines

A20 (H2K^d^) lymphoma cell line was purchased from American Type Culture Collection (Manassas, VA). Luciferase-expressing A20 cells were generated by a lentiviral system. Briefly, the luciferase gene was ligated into a lentiviral plasmid (pRRL-Venus, provided by Dr. Yun Zhao, Soochow University, Suzhou, China). The luciferase-expressing lentivirus generated from 293T cells (ATCC, Manassas, VA) was used to infect A20 cells to generate the A20-luc^+^/yfp cells. Stable luciferase-expressing A20 cells were sorted by flow cytometry (BD FACS Aria III, BD Bioscience, San Jose, CA), and cultured with RPMI 1640 supplemented with 10% FBS (both from Hyclone, Marlborough, MA).

### Murine GVL and aGVHD Models

GVL model: BALB/c recipient mice received lethal TBI (750cGy: 2 doses of 375cGy with 4 h interval) from a ^137^Cs source. Three hours later, 5×10^6^ BMCs from WT or TCR-δ^-/-^ C57BL/6 mice plus 1×10^6^ A20 lymphoma cells or 5×10^6^ A20- luc+/yfp cells were intravenously injected into lethally irradiated BALB/c recipients. The survival was monitored and the body weights of recipients were assessed every other day.

aGVHD model: BALB/c recipient mice received lethal TBI (750cGy: 2 doses of 375cGy with 4 h interval) from a ^137^Cs source. Three hours later, 1×10^7^ BMCs plus 5×10^6^ splenocytes from WT or TCR-δ ^-/-^ C57BL/6 mice were intravenously injected into lethally irradiated BALB/c recipient. The survivals were observed, and the body weights and clinical scores were assessed every two or three days. The severity of aGVHD was assessed with a clinical GVHD scoring system as described by Cooke et al. in a blinded fashion. The degree of systemic GVHD was assessed by the sum of changes in five clinical parameters: weight loss, posture (hunching), activity, fur texture, and skin integrity (30).

### In Vitro Expansion and Adoptive Transfer of γδT/Vγ1/Vγ4 Cells

Firstly, 10 µg/ml anti-mouse TCRγδ (GL3), Vγ1 (2.11) or Vγ4 (UC3) antibody was coated in 6-well plates (1 ml/well) overnight at 4°C. The coated plates were washed gently three times with PBS. The splenocytes from CD45.1-TCR-β^-/-^ mice were seeded into the antibody-coated plates (2.5 × 10^6^ cells/ml) and cultured for seven days in the presence of rhIL-2 (100 IU/ml, Miltenyi Biotec, Bergisch Gladbach, Germany). The purity of expanded γδT cells was more than 95%. 1×10^7^ γδT/Vγ1/Vγ4 cells were adoptively transferred into the recipient mice on day 0 of allo-HSCT. The purified anti-mouse TCRγδ antibody was purchased from BD Bioscience (San Diego, CA). The purified anti-mouse Vγ1 (2.11) and Vγ4 (UC3) antibodies were purchased from Sungene Bio-technological company (Tianjin, China).

### Depletion of Vγ1 or Vγ4 γδT Cells In Vivo

To deplete Vγ1 or Vγ4 subset, polyclonal hamster IgG, purified anti-mouse Vγ1 (2.11) or purified anti-mouse Vγ4 (UC3) antibody (all from BioXcell, West Lebanon, NH) was intraperitoneally injected into the recipient mice (100 µg/200 µl/mouse) once weekly. The depletion efficiency was confirmed by flow cytometry (**Figure S2**).

### Bioluminescent Imaging

5×10^6^ luciferase-expressing A20 lymphoma cells were adoptively transferred into lethally irradiated BALB/c mice along with BMCs derived from WT or TCR-δ^-/-^C57BL/6 mice on the day of transplantation. For measuring leukemia growth by bioluminescence imaging (BLI), mice were anesthetized by injecting *i.p.* 10% chloral hydrate then injected *i.p.* with 100 μl of 150 μg/ml D-luciferin (Gold Biotechnology, St. Louis, MO). Five minutes later, mice were imaged using Xenogen, IVIS 100 Bioluminescent Imaging System (Caliper Life Sciences, Hopkinton, MA) to determine the level of leukemia burdens.

### Cytotoxicity Assay

Cytotoxicity assay was carried out using a cytotoxicity detection kit (LDH) (Roche, Basel, Switzerland). Expanded γδT/Vγ1/Vγ4 cells or isolated CD8^+^T cells from the spleen of recipient mice were obtained and cocultured with A20 cells at different E:T ratios for 6 hours. The killing capability was assessed according to the manufacturer’s protocol. The percentage of cytotoxicity at each E:T ratio was calculated using the following formula: percentage of cytotoxicity = (experimental − effector spontaneous − target spontaneous)/(target maximum − target spontaneous) × 100%.

### Flow Cytometry

Single cell suspensions from spleens, livers, lungs, and IELs were obtained according to the methods previously described (31) and analyzed by using flow cytometry. The antibodies used for FACS staining: PE-CF594-anti-mouse-CD3e (145-2C11), APC-Cy7-anti-mouse-CD45.1 (A20), Percp-Cy5.5-anti-mouse-TCRγδ (GL3), Alexa Fluor700-anti-mouse-NK1.1 (PK136) were purchased from BD Bioscience (San Diego, CA); purified anti-mouse-CD16/32 (**93**), PE/APC-anti-mouse IL-17A (TC11-18H10.1), PE-anti-mouse-NKG2D (C7), FITC-antimouse-NKG2D (C7), PE-anti-mouse-IFN-γ (XMG1.2), PE/Cy7-anti-mouse TNF-α (MP6-XT22), FITC-anti-mouse-CD69(H1.2F3), PE-anti-mouse-CD62L(MEL-14), APC-anti-mouse-CD44 (BJ18) were purchased from Biolegend (San Diego, CA); PE-Cy7-anti-mouse- Granzyme B (NGZB), APC-anti-mouse-Perforin (eBioOMAK-D) were purchased from eBioscience (San Diego, CA). FITC-anti-mouse-Vγ1, APC-anti-mouse-Vγ4 were purchased from Sungene Bio-technological Company (Tianjin, China). Flow cytometric analysis were performed using a FACS Canto II (BD Biosciences, San Jose, CA) or NovoCyte (ACEA Biosciences, San Diego, CA) flow cytometer and analyzed by the Flowjo software (Tree Star, Ashland, OR).

### Statistical Analysis

One-way ANOVA was used to determine statistically significant differences among more than two experimental groups. The unpaired Student t-test was used to determine statistically significant differences between the two experimental groups. Data were analyzed using GraphPad Prism 5 software for Windows (GraphPad Software, San Diego, CA). *P*-value <0.05 was considered statistically significant (*), the significance levels are marked as *p <0.05, ** *p*<0.01, *** *p*<0.001 and **** *p*<0.0001.

## Results

### Donor γδT Cells Exert GVL Effect During allo-HSCT

**Figure 1:**
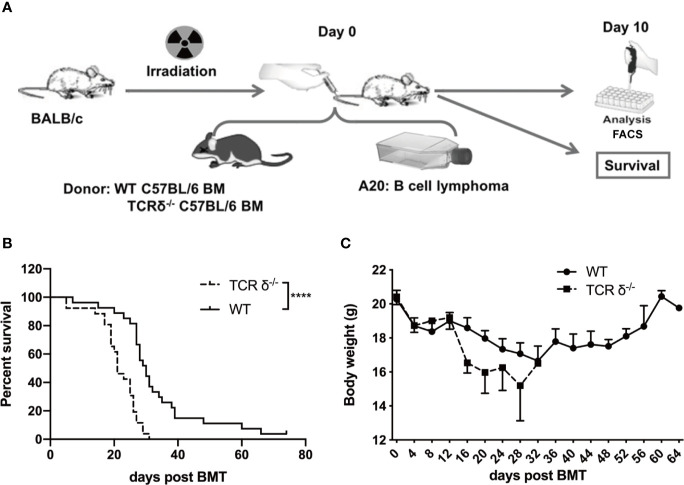
The GVL effect of donor-derived γδT cells in allo-HSCT. **(A)** The experimental design of murine GVL model. Recipient mice (BALB/c) were lethally irradiated and received A20 lymphoma cells (1 × 10^6^ cells/mouse, *iv*.) plus BMCs (5 × 10^6^ cells/mouse, *iv*.) from WT or TCRδ^‐/-^ mice. The phenotypes of immune cells from spleens and livers of recipient mice were examined on day 10 post allo-HSCT. The survival **(B)** and the body weight **(C)** of recipients were observed and measured (n = 26–27 mice per group). Results shown **(B, C)** were pooled from 3 independent experiments. The graph **(C)** displays mean ± SEM. Significance was determined by log-rank (Mantel-Cox) survival test **(B)** and unpaired 2-tailed Student’s t tests **(C)**. ****p < 0.0001.

To investigate the role of donor γδT cells during allo-HSCT, we established a murine GVL model. BALB/c mice were lethally irradiated and received bone marrow cells (BMCs, 5×10^6^ cells/mouse, *iv*.) from WT or γδT deficient (TCRδ^-/-^) C57BL/6 donor mice. A20 cells (1 × 10^6^ cells/mouse, *iv*.) were injected into the recipients intravenously on the day of transplantation (**Figure 1A**). The survival of the recipient mice was monitored and weighed every other day. The results showed that in the absence of donor γδT cells, the survival and body weights of the recipient mice were significantly reduced (**Figures 1B**), suggesting that donor-derived γδT cells could enhance GVL effect during allo-HSCT.

### Donor γδT Cells Are Essential for the Production of IFN-γ in CD8^+^ T Cells

**Figure 2:**
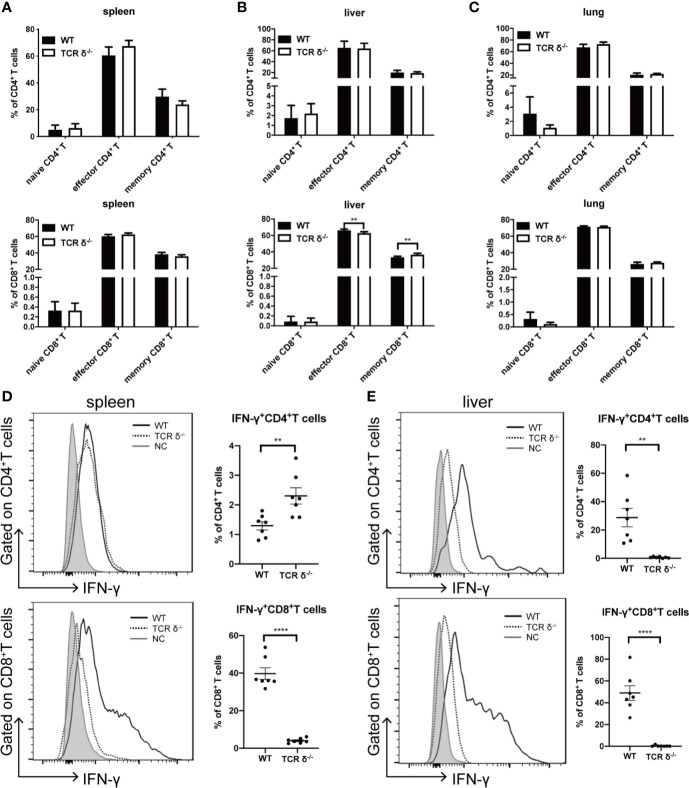
Activation phenotypes and IFN-γ production of CD4^+^ and CD8^+^ T cells in the recipients of WT or TCRδ^-/-^ grafts after allo-HSCT. The allo-HSCT was performed as described in **Figure 1A**. The lymphocytes were isolated from host spleen or liver on day 10 post allo-HSCT and analyzed by flow cytometry. Percentages of naïve, effector and memory CD4^+^ and CD8^+^ T cells in spleen **(A)**, liver **(B)**, and lung **(C)** were shown. The production of IFN-γ by CD4^+^ and CD8^+^ T cells in the spleen **(D)** and liver **(E)** post allo-HSCT were examined (NC-Negative control: grey shaded, WT: solid line, TCRδ^-/-^: dotted line). All data are representative of at least 3 independent experiments with n = 7 mice per group. All graphs display mean ± SEM. Significance was determined by unpaired 2-tailed Student’s t tests. **p < 0.01, ****p < 0.0001.

Other than direct cytotoxicity against tumor cells, donor γδT cells may have anti-leukemia effect by regulating αβT cell functions (32). To explore the regulatory role of donor γδT cells *in vivo*, we examined the immune phenotypes of T lymphocytes on day 10 post allo-HSCT in mice receiving either WT or TCR δ^-/-^ BMs (5×10^6^ cells/mouse, *iv*.) together with A20 cells (1 × 10^6^ cells/mouse, *iv*.) (**Figures 2A–C**). By CD62L and CD44 expression, naïve, effector, and memory subsets of CD4^+^ and CD8^+^T subsets from the spleen, liver, and lung were examined. The results showed that there was no obvious difference in the activation of CD4^+^ or CD8^+^ T cells between the mice receiving WT BMCs and those receiving TCR δ^-/-^ BMCs.

IFN-γ is critical in mediating anti-leukemia activity of T lymphocytes (33, 34). We then examined the IFN-γ production by both CD4^+^ and CD8^+^ T cells in the spleen and liver of the recipient mice (**Figures 2D, E**). CD8^+^ T cells were the main IFN-γ producers, and the results showed that the capability of CD8^+^ T cells producing IFN-γ was severely impaired in the absence of donor γδT cells. The percent of IFN-γ-producing CD8^+^ T cells decreased from 39.7 to 4.0% in the spleen and from 49.0 to 0.4% in the liver. To exclude the possibility of intrinsic low IFN-γ production by CD8^+^ T cells in TCR-δ^-/-^ mice, we examined IFN-γ production by both CD4^+^ and CD8^+^ T cells in the spleen and liver of naïve WT and TCR-δ^-/-^ mice (**Figures S1A, B**). The results showed that there was no difference in the percent of CD4^+^ and CD8^+^ T cells, or the IFN-γ-producing cells between WT and TCR-δ^-/-^ mice. To investigate whether the different IFN-γ production by CD8^+^ T cells in the recipients was affected by the changes in regulatory T cells, we examined the proportion of regulatory T cells in the spleen and liver of the host mice that received BMCs from WT or TCR-δ^-/-^ mice. We found that there was no difference in the percentages of regulatory T cells in the spleen and liver of the recipient mice receiving WT or TCR-δ^-/-^ BMCs (**Figure S1C**). Thus, donor γδT cells could exert GVL effect *via* regulating IFN-γ production by CD8^+^ T cells in allo-HSCT without affecting regulatory T cells.

### Donor Vγ4 γδT Cells Are the Main Cell Subset Mediating GVL Effect

**Figure 3:**
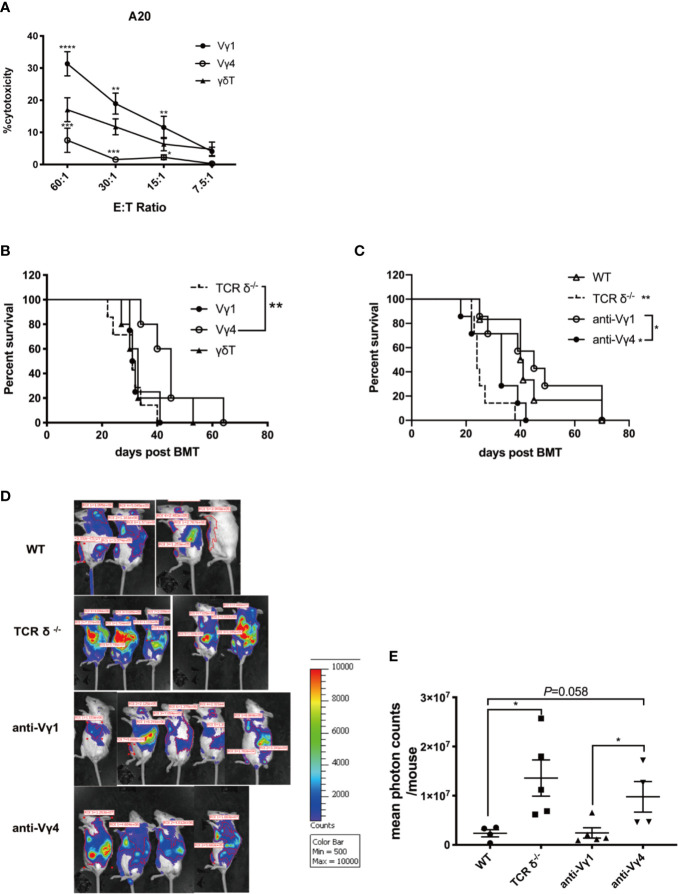
The GVL effect of Vγ1 and Vγ4 γδT cell subsets. **(A)** The cytotoxicity of Vγ1, Vγ4, and total γδT cells expanded from CD45.1-TCR-β^-/-^ mouse against A20 cells *in vitro*. **(B)** The survival of recipient mice that received Vγ1, Vγ4, or total γδT cell infusion. Recipient mice were lethally irradiated and received A20 lymphoma cells (1 × 10^6^ cells/mouse, *iv*.) plus BMCs (5 × 10^6^ cells/mouse, *iv*.) from TCRδ^‐/-^ mice. Vγ1, Vγ4, or total γδT cells (1 × 10^7^ cells/mouse, *iv*.) were adoptively transferred into the recipients on day 0. **(C)** The survival of recipient mice that were depleted of Vγ1 or Vγ4 subsets. Recipient mice were lethally irradiated and received A20 lymphoma cells (1 × 10^6^ cells/mouse, *iv*.) plus BMCs (5 × 10^6^ cells/mouse, *iv*.) from WT donor mice. Anti-Vγ1 or anti-Vγ4 antibody (100 µg/200 µl/mouse, *ip*.) was administered once a week for 4 weeks intraperitoneally. IgG (100 µg/200 µl/mouse, *ip*.) was injected as control. **(D)** The BLI of recipients that were depleted of Vγ1 or Vγ4 cells, together with WT and TCRδ^-/-^ donor controls on day 14 after allo-HSCT. Recipient mice were lethally irradiated and received A20- luc+/yfp lymphoma cells (5 × 10^6^ cells/mouse, *iv*.) plus BMCs (5 × 10^6^ cells/mouse, *iv*.) from WT donor mice. Anti-Vγ1 or anti-Vγ4 antibody (100 µg/200 µl/mouse, *ip*.) was administered once a week for 4 weeks intraperitoneally. IgG (100 µg/200 µl/mouse, *ip*.) was injected as control. **(D)** Quantitative analysis of the imaging results. All data are representative of at least 3 independent experiments. There were at least 5 mice per group for B and **(C)** For panel D and E, there were 4 or 5 mice per group. All graphs display mean ± SEM. Significance was determined by log-rank (Mantel-Cox) survival test **(B, C)** and one-way ANOVA test **(A, E)**. *p < 0.05, **p < 0.01, ***p < 0.001, ****p < 0.0001.

There are two major γδT cell subsets in the mouse periphery tissues, Vγ1 and Vγ4 cells. These two subsets can have different functions in various diseases (10). To investigate the roles of donor Vγ1 and Vγ4 cell subsets in mediating GVL effect post allo-HSCT, we first compared the cytotoxicity of the two cell subsets against A20 cells *in vitro* (**Figure 3A**). We found that Vγ1 cells exhibited a significantly higher level of cytotoxicity against A20 cells compared with Vγ4 cells or total γδT cells. To investigate whether Vγ1 cells are also the main cell subset mediating GVL effect *in vivo*, we adoptively transferred *in vitro* expanded Vγ1, Vγ4, or γδT cells (1 × 10^7^ cells/mouse, *iv*.) and BMCs from TCR-δ^-/-^ mouse (5 × 10^6^ cells/mouse, *iv*.), as well as A20 cells (1 × 10^6^ cells/mouse, *iv*.) on day 0 of allo-HSCT (**Figure 3B**). The results showed Vγ4 cell adoptive transfer significantly prolonged the survival of the recipients that received no γδT cell infusion, while Vγ1 or total γδT cell infusion had no significant effect on their survival, suggesting Vγ4 cells were the main cell subset mediating GVL effect during allo-HSCT.

To further confirm the GVL function of Vγ4 cells, we depleted the Vγ1 or Vγ4 cells using specific anti-Vγ1 or anti-Vγ4 antibodies in WT BMC recipient mice (**Figure 3C**). Anti-Vγ1 or anti-Vγ4 antibodies (100 µg/200 µl/mouse, *ip*.) were administered once a week for 4 weeks. The depletion efficiency of either cell subset was confirmed by flow cytometry (**Figure S2**). The results showed that the depletion of Vγ4 cells significantly reduced the survival of the recipients compared to WT group, while the depletion of Vγ1 cells did not affect the survival of allo-HSCT recipients. To visualize and quantify leukemia growth in the hosts, we performed bioluminescent imaging by establishing a murine leukemia model using A20-luc cells (**Figure 3D**). The results showed that the depletion of Vγ1 cells displayed a comparable tumor burden to that of WT group. However, the depletion of Vγ4 cells resulted in a higher tumor burden than WT or anti-Vγ1 group, which was comparable to that of TCR-δ^-/-^ BMC recipients (**Figures 3D, E**). These results demonstrated that Vγ4 cells were the major γδT cell subset mediating GVL effect during allo-HSCT.

### The GVL Function of Vγ4 cells Is Partially Dependent on IL-17A Production

**Figure 4:**
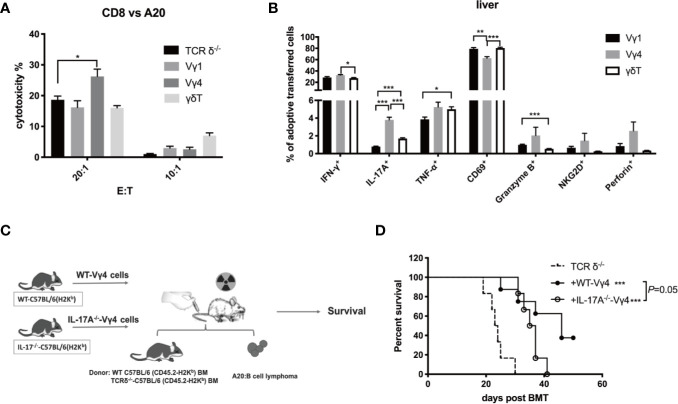
The enhanced GVL effect of Vγ4 cells was partially dependent on the production of IL-17A. Recipient mice were lethally irradiated and received A20 lymphoma cells (1 × 10^6^ cells/mouse, *iv*.) plus BMCs (5 × 10^6^ cells/mouse, *iv*.) from TCRδ^‐/-^ mice. Vγ1, Vγ4, or total γδT cells (1 × 10^7^ cells/mouse, *iv*.) from CD45.1-TCRβ^-/-^ mouse were adoptively transferred into the recipients on day 0. **(A)** The CD8^+^ T cells were isolated from the recipient mice (purity >99%) that received the A20 cells (1 × 10^6^ cells/mouse, *iv*.) plus BMCs (5 × 10^6^ cells/mouse, *iv*.) from TCRδ^‐/-^ mice with or without the adoptive transfer of Vγ1, Vγ4, or γδT cells (1 × 10^7^ cells/mouse, *iv*.) on day 7 post allo-HSCT. Cytotoxicity against A20 cells (at E:T ratio of 20:1 and 10:1) was measured by LDH assay. **(B)** The activation marker expression and cytokine production of adoptively transferred Vγ1, Vγ4, or γδT cells in the liver were examined by flow cytometry on day 7 post allo-HSCT. **(C)** Experimental design: BALB/c recipients were lethally irradiated and received A20 lymphoma cells cells (1 × 10^6^ cells/mouse, *iv*.) and BMCs (5 × 10^6^ cells/mouse, *iv*.) from WT or TCRδ^‐/-^ mice. Vγ4 cells (1 × 10^7^ cells/mouse, *iv*.) expanded from WT or IL-17A^-/-^ mouse were adoptively transferred into recipients on day 0. **(D)** The survival of recipients that received WT-Vγ4 or IL-17A^-/–^Vγ4 cells. All data are representative of at least 3 independent experiments with n ≥5 mice per group. All graphs display mean ± SEM. Significance was determined by one-way ANOVA test (A–B) and log-rank (Mantel-Cox) survival test **(D)**. *p < 0.05, **p < 0.01, ***p < 0.001.

To investigate the mechanism of Vγ4 cells mediating GVL function *in vivo*, we first compared the phenotypes of *in vitro* expanded Vγ1, Vγ4, and γδT cells (**Figures S3A–G**). Flow cytometry analysis revealed that the percentage of IL-17A-producing cells was higher in Vγ4 cells than in Vγ1 and total γδT cells. We then established murine GVL model and adoptively transferred donor Vγ1, Vγ4, or γδT cells (1 × 10^7^ cells/mouse, *iv*.) derived from CD45.1-TCR-β^-/-^ mice together with BMCs (5 × 10^6^ cells/mouse, *iv*.) from TCRδ^-/-^ mice and A20 cells (1 × 10^6^ cells/mouse, iv.). CD8^+^ T cells from spleens of the recipient mice were isolated on day 7 post-transplantation and the cytotoxicity of CD8^+^ T cells against A20 cells was measured (**Figure 4A**). The results showed that CD8^+^ T cells from the mice receiving Vγ4 cells displayed increased cytotoxicity against A20 cells compared to the mice receiving no adoptive transfer. The total number of CD8^+^ T cells in the spleen and liver of the recipient mice showed no difference among different groups (**Figure S3H**). Flow cytometry results demonstrated that the percentage of IL-17A-producing cells was significantly higher in adoptively transferred Vγ4 cells than in Vγ1 or γδT cells in the liver of the recipient mice (**Figure 4B**), which is consistent with their phenotypes *in vitro*. IL-17A has been shown to promote anti-tumor T cell response in murine models and human tumors (35, 36). It might be the effector molecule mediating the regulatory function of Vγ4 cells.

To confirm whether the GVL function of Vγ4 cells is mediated by IL-17A, we expanded Vγ4 cells from WT or IL-17A^-/-^ mice (both of C57BL/6 background) and adoptively transferred them (1 × 10^7^ cells/mouse, *iv*.) into recipient mice of TCR-δ^-/-^ BMCs (5 × 10^6^ cells/mouse, *iv*.) and A20 cells (1 × 10^6^ cells/mouse, *iv*.) (**Figure 4C**). Although the infusion of either WT-Vγ4 or IL-17A^-/–^Vγ4 cells could prolong the survival of the hosts, the recipients of WT-Vγ4 cells exhibited slightly better survival compared to the recipients of IL-17A^-/–^Vγ4 cells (*p* = 0.05, **Figure 4D**). These results suggest that the GVL effect of Vγ4 cells could be partially mediated by IL-17A.

### Donor γδT Cells Mitigate aGVHD During allo-HSCT

**Figure 5:**
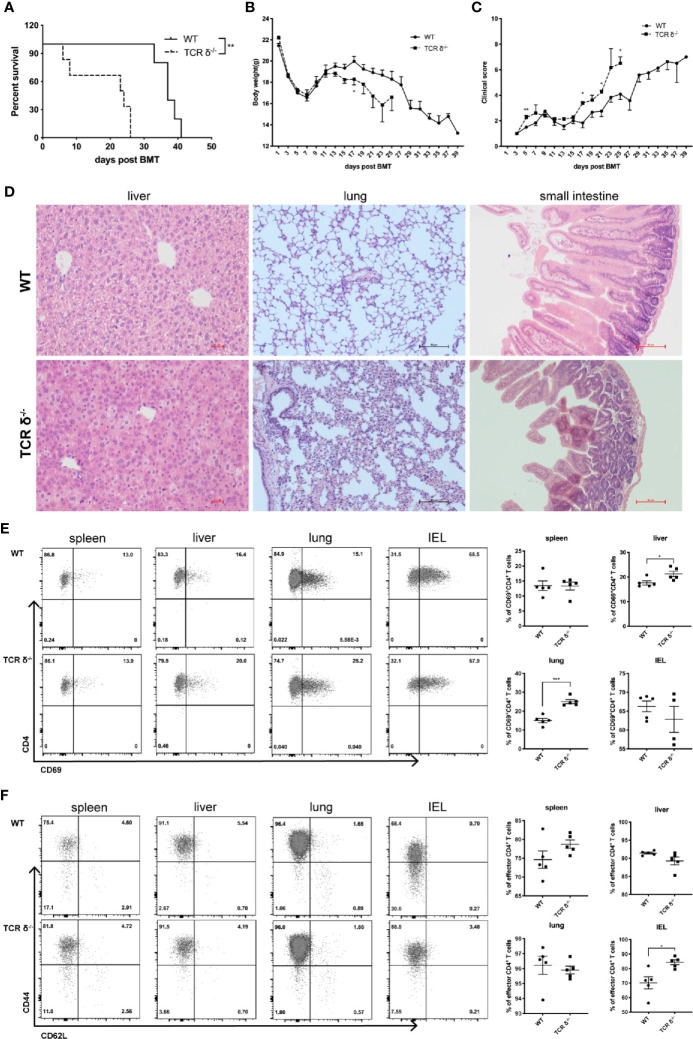
Donor-derived γδT cells could mitigate aGVHD during allo-HSCT. Recipient mice (BALB/c) were lethally irradiated and given splenocytes (5 × 10^6^ cells/mouse, *iv*.) plus BMCs (1 × 10^7^ cells/mouse, *iv*.) from WT or TCR-δ^-/-^ C57BL/6 donor mouse to establish murine aGVHD model after allo-HSCT. Survival of recipients **(A)**, body weight changes **(B)**, and clinical scores **(C)** were monitored over time. **(D)** Histopathology of livers, lungs and small intestines of the recipients of WT or TCR δ^-/-^ grafts on day 7 post transplantation. The activation phenotype of lymphocytes was examined on day 7 post allo-HSCT. **(E)** The percentages of activated CD4^+^ T cells in the spleen, liver, lung and IEL of the recipients. **(F)** The percentages of effector CD4^+^ T cells in the spleen, liver, lung and IEL of the recipients. All data are representative of at least 3 independent experiments with n = 4–6 mice per group. All graphs display mean ± SEM. Significance was determined by log-rank (Mantel-Cox) survival test **(A)** and unpaired 2-tailed Student’s t tests (B-E). *p < 0.05, **p<0.01, ***p < 0.001.

aGVHD is one of the major complications post allo-HSCT causing patients’ mortality. Generally, aGVHD is induced by αβT cells that are also critical for GVL function (4). To determine whether donor γδT cells could induce aGVHD while promoting GVL effects, we established a murine aGVHD model by using BMCs (1 × 10^7^ cells/mouse, *iv*.) and splenocytes (5 × 10^6^ cells/mouse, *iv*.) from WT or TCR-δ^-/-^ mice (C57BL/6) (**Figure 5A**). The results showed that the deficiency of γδT cells in donor grafts resulted in accelerated aGVHD-related death in recipient mice. The recipients that received TCR-δ^-/-^ grafts displayed lower body weight and higher GVHD clinical scores compared with the WT group (**Figures 5B, C**). In addition, we performed histopathology with livers, lungs, and small intestines from the recipients on day 7 post-transplantation. Compared with the WT recipients, the TCR-δ^-/-^ recipient mice displayed much more severe tissue damage in the livers, lungs, and small intestines known as the typical characteristics of aGVHD, including the damage of parenchymal hepatic cells and pulmonary alveoli, the infiltration of lymphocytes, incomplete intestinal villus epithelial structure, and epithelial cell shedding (**Figure 5D**). These results indicated that donor γδT cells could mitigate aGVHD during allo-HSCT.

To investigate whether αβT cell activation could be affected by donor γδT cells in the murine aGVHD model, we examined the immune phenotypes of αβT cells from aGVHD target organs. In the absence of donor γδT cells, percent of activated CD4^+^ T cells was significantly increased in the liver and lung, while the changes were not significant in the spleen or intestinal intraepithelial lymphocytes (IELs) (**Figure 5E**). Moreover, the percentage of CD44^+^CD62L^-^ effector CD4^+^T cells displayed a significant increase in the IELs and a trend of increase in the spleen in the TCR-δ^-/-^ graft recipients compared with WT recipients (**Figure 5F**). These results suggest that donor γδT cells may suppress aGVHD by inhibiting CD4^+^ T cell activation.

### Donor Vγ4 γδT Cells Are the Main Cell Subset Mitigating aGVHD

**Figure 6:**
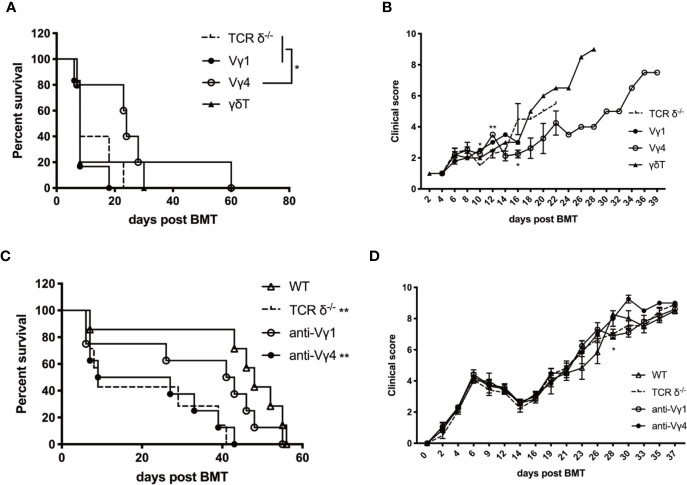
Donor Vγ4 γδT cells are the main cell subset mitigating aGVHD. The survival **(A)** and clinical scores **(B)** of aGVHD recipients that received adoptively transferred Vγ1, Vγ4, or total γδT cells. BALB/c mice were lethally irradiated and received splenocytes (5 × 10^6^ cells/mouse, *iv*.) plus BMCs (1 × 10^7^ cells/mouse, *iv*.) from TCRδ^‐/-^ mice. Vγ1, Vγ4, or total γδT cells (1 × 10^7^ cells/mouse, *iv*.) were infused into recipients on day 0. The survival **(C)** and clinical score **(D)** of aGVHD recipients that were depleted of Vγ1 or Vγ4 cells. BALB/c mice were lethally irradiated and received splenocytes (4 × 10^6^ cells/mouse, iv.) plus BMCs (1 × 10^7^ cells/mouse, *iv*.) from WT donors. Anti-Vγ1 or anti-Vγ4 antibody (100 µg/200 µl/mouse) was administered into the recipients once a week for 3 weeks intraperitoneally. IgG (100 µg/200 µl/mouse, *ip*.) was injected as controls. All data are representative of at least 3 independent experiments with n ≥5 mice per group. All summary graphs display mean ± SEM. Significance was determined by log-rank (Mantel-Cox) survival test **(A, C)** and one-way ANOVA **(B, D)**. *p < 0.05, **p < 0.01.

To investigate which γδT cell subset plays the protective role in aGVHD, we adoptively transferred the *in vitro* expanded Vγ1, Vγ4, or total γδT cells (1 × 10^7^ cells/mouse, *iv*. from TCR-β^-/–^C57BL/6 mice) in the murine aGVHD model described before (**Figures 6A, B**). Vγ4 cell infusion significantly prolonged the survival of aGVHD recipients of TCR-δ^-/-^ grafts and reduced the severity of aGVHD symptoms, while Vγ1 or total γδT cell infusion had no effect on the progression of aGVHD. These results suggest Vγ4 γδT cells could be the main cell subset mediating the protective effect of aGVHD.

To further confirm this finding, Vγ1 or Vγ4 γδT cells were depleted with specific anti-Vγ1 or anti-Vγ4 antibodies (100 µg/200 µl/mouse, *ip*.) in the WT recipients (**Figures 6C, D**). Depletion of Vγ4 cells aggravated the progress of aGVHD and the survival was similar to that of the TCR-δ^-/-^ recipients, while Vγ1 depletion exhibited no effect on aGVHD progression in the WT recipients. These results indicated that donor Vγ4 cells were the main γδT cell subset mitigating aGVHD during allo-HSCT.

## Discussion

γδT cells have been reported to reconstitute faster than αβT cells after allo-HSCT (13), thus might play an important role in modulating GVL and aGVHD at the early stage of allo-HSCT. γδT cells and NK cells share a series of features, including the expression of surface receptors and non-MHC-restricted recognition (22). Donor NK cell infusion can promote engraftment, enhance GVL effect and suppress aGVHD after allo-HSCT (37). In the current study, we found that donor γδT cells could also promote GVL effect and mitigate aGVHD during allo-HSCT. Further analysis revealed that Vγ4 γδT cells were the main cell subset mediating both functions by regulating CD4^+^ and CD8^+^ αβT cell responses.

γδT cells have been shown to have direct cytotoxicity against tumor cells. Human γδT cells can directly kill CML blasts and other tumor cells (15, 38, 39). *In vitro* expanded human Vγ9Vδ2 T cells can efficiently kill EBV-transformed autologous lymphoblastic B cell lines (16). The adoptive transfer of these expanded human Vγ9Vδ2 T cells significantly prevents disease progression in humanized mice. γδT cells also display direct cytotoxicity against solid tumors, such as melanoma, prostate cancer, breast cancer, and lung carcinomas (23, 40–42). γδT cells exert the direct anti-tumor effect by the engagement of surface receptors, including γδTCR and NKG2D. The expanded donor γδT cells in our experimental system also exhibited direct cytotoxicity against A20 cells. However, this *in vitro* killing capacity was not associated with the *in vivo* anti-leukemia activity of the γδT cell subsets we examined. Vγ4 γδT cells showed a lower level of cytotoxicity *in vitro* but superior GVL effect *in vivo* compared to Vγ1 γδT cells, suggesting the immune regulatory role of γδT cells may be more critical than their direct killing capacity in regulating GVL effect *in vivo* after allo-HSCT.

Other than direct cytotoxicity against tumor cells, γδT cells can also function as antigen presenting cells to stimulate adaptive immune responses. Human Vγ9Vδ2 T cells expanded *in vitro* can present exogenous soluble protein epitopes *via* MHC class I complexes to antigen-specific CD8^+^ αβT cells (32). Due to their early reconstitution after allo-HSCT, γδT cells may serve as antigen presenting cells at the early stage of immune reconstitution to activate leukemia-specific CD8^+^ T cell response. We found that IFN-γ production in CD8^+^ T cells was severely impaired in the absence of donor γδT cells post allo-HSCT. By using IL-17A-deficient donor Vγ4 γδT cells, we also demonstrated that the GVL effect mediated by Vγ4 γδT cells was partially dependent on IL-17A. The role of IL-17A-producing γδT cells in tumor development is controversial and could be tumor model-dependent. They are found to promote tumor growth and metastasis in both mice and humans (43). Vγ4 γδT cells in the liver can enhance the development of murine hepatocellular carcinoma by producing chemokines that recruit MDSCs in the tumor microenvironment (19). Consistently, IL-17-producing γδT cell infiltration is positively correlated with the severity of human colorectal carcinoma (44). However, there are also studies showing that the anti-tumor CD8^+^ T cell response can be facilitated by IL-17-producing γδT cells (35, 45). Therefore, the function of IL-17-producing γδT cells could be tumor type- and environment-dependent. In the current study, we discovered that IL-17A produced by donor Vγ4 γδT cells might be involved in promoting the GVL effect after allo-HSCT. Further studies are needed to investigate the mechanism of such an effect mediated by IL-17A.

Interestingly, our previous study showed that IL-17A was protective in murine aGVHD models by modulating CD4^+^ T cell responses (46). In fact, donor Vγ4 γδT cells, which produce higher levels of IL-17A than other γδT cell subsets, mitigated aGVHD in the murine model of allo-HSCT. Donor γδT cells significantly inhibited CD4^+^ T cell activation, which is the main cellular event for aGVHD responses. However, one study showed that the depletion of donor γδT cells prevented aGVHD during allo-HSCT (47). A recent study reported donor γδT cells alleviated aGVHD when the administration of αβT cells was delayed for two weeks and the mitigation of aGVHD by donor γδT cells occurred only at high doses (25). This low efficacy of donor γδT cell infusion in mitigating aGVHD could be due to the heterogeneity of the *in vitro* expanded γδT cells. We demonstrated by using TCRδ^-/-^ donors that donor γδT cells are critical in mitigating aGVHD during allo-HSCT. However, only infusion of Vγ4 γδT cells exhibited prolonged survival in recipient mice, while adoptively transfer of total γδT cells had no effect on the progression of aGVHD, which is consistent with the previous study. Nevertheless, the detailed mechanism of Vγ4 γδT cells mitigating aGVHD warrants further studies.

Different subsets of γδT cells have been reported to have different, even opposite roles in various diseases. In B16 melanoma model, activated CD44^high^ Vγ4 cells but not Vγ1 cells exert dominant anti-tumor function by producing IFN-γ and perforin (17). These two subsets were also reported to play distinct and opposing functions in the EAE model. Vγ4 cells exacerbate disease symptoms by producing IL-17A, while Vγ1 subset plays a protective role by secreting CCR5 ligands to regulate the Treg-Th17 balance (48). In human studies, high total γδT cell numbers after HSCT are associated with a favorable clinical outcome but not with aGVHD incidence (49). A recent study in 105 allo-HSCT recipients showed that the higher proportions of CD8^+^ γδT cells in the graft were associated with an increased incidence of aGVHD, while high proportions of CD27^+^ γδT cells had a trend of an inverse association with the relapse (50). Although there are studies indicating the links between the murine Vγ γδT subsets and human Vδ γδT subpopulations, how the functions of the murine γδT cell subsets can be correlated with human γδT cell populations needs further investigations.

Disease relapse and aGVHD are the main complications leading to the failure of allo-HSCT. Novel strategies are urgently needed to prevent aGVHD while preserving or promoting GVL effect. Our findings provide evidence supporting the notion that donor γδT cell infusion could be a potentially effective therapeutic strategy to enhance GVL and mitigate aGVHD during allo-HSCT.

## Data Availability Statement

All datasets presented in this study are included in the article/**Supplementary Material**.

## Ethics Statement

The animal study was reviewed and approved by Institutional Laboratory Animal Care and Use Committee of Soochow University and National University of Singapore.

## Author Contributions

HL and DW designed the study. YS, YZ, BH, YL, DL, and ZJ performed the experiments. YS, YZ, BH, DL, and HL analyzed the results and wrote the manuscript. ZY and CD provided essential materials and suggestions for data presentation. All authors contributed to the article and approved the submitted version.

## Funding

This work has been supported by National Key R&D Program of China (2017YFA0104502, 2016YFC0902800), National Natural Science Foundation of China (81571556, 81730003, and 81470346), Priority Academic Program Development of Jiangsu Higher Education Institutions (PAPD), and start-up grant of National University of Singapore.

## Conflict of Interest

The authors declare that the research was conducted in the absence of any commercial or financial relationships that could be construed as a potential conflict of interest.
