# Supplementary material for: Donor γδT Cells Promote GVL Effect and Mitigate aGVHD in Allogeneic Hematopoietic Stem Cell Transplantation
Source: Front Immunol. 2020 Oct 16;11:558143. doi: 10.3389/fimmu.2020.558143 (PMC7596318; doi:10.3389/fimmu.2020.558143)
Supplement: Supplementary file 1 [file DataSheet_1.pdf]

Supplementary Material

Donor γδT cells promote GVL effect and mitigate aGVHD in allogeneic hematopoietic stem cell transplantation

Yuan Song[1†], Ying Zhu[1†], Bo Hu[2*†], Yonghao Liu [1], Dandan Lin[2], Ziqi Jin [2], Zhinan Yin[3,4], Chen Dong[5], Depei Wu[2*] and Haiyan Liu[1*]

Corresponding author:

Dr. Haiyan Liu, Email: micliuh@nus.edu.sg

Or

Dr. Depei Wu, Email: wudepei@suda.edu.cn

Or

Dr. Bo Hu, Email: hubo82428@163.com

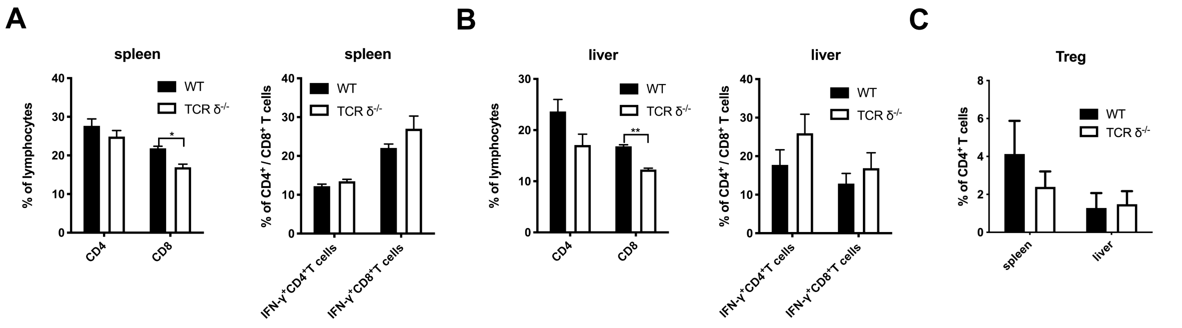

Supplemental Figure 1

Supplemental Figure 1. IFN-γ production by CD4$^+$ and CD8$^+$ T cells in naïve WT and TCR-δ$^{-/-}$ C57BL/6 mice. The percentages of CD4$^+$ and CD8$^+$T cells and the percentage of IFN-γ-producing CD4$^+$ and CD8$^+$ T cells from the spleen (A) and liver (B) of naïve WT or TCR-δ$^{-/-}$ mouse. (C) The percentage of Treg cells in the spleen and liver from the recipients. Recipient mice (BALB/c) were lethally irradiated and received A20 lymphoma cells (1×10^6/mouse, iv.) plus BM cells (5×10^6/mouse, *iv.*) from WT or TCRδ$^{-/-}$ mice. 10 days later, the percentage of Treg cells in the spleen and liver of hosts was detected by flow cytometry. Data shown are the representative of two independent experiments. All graphs displayed mean ± SD. *$p < 0.05$, **$p < 0.01$ determined by two-tailed unpaired Student's t-test.

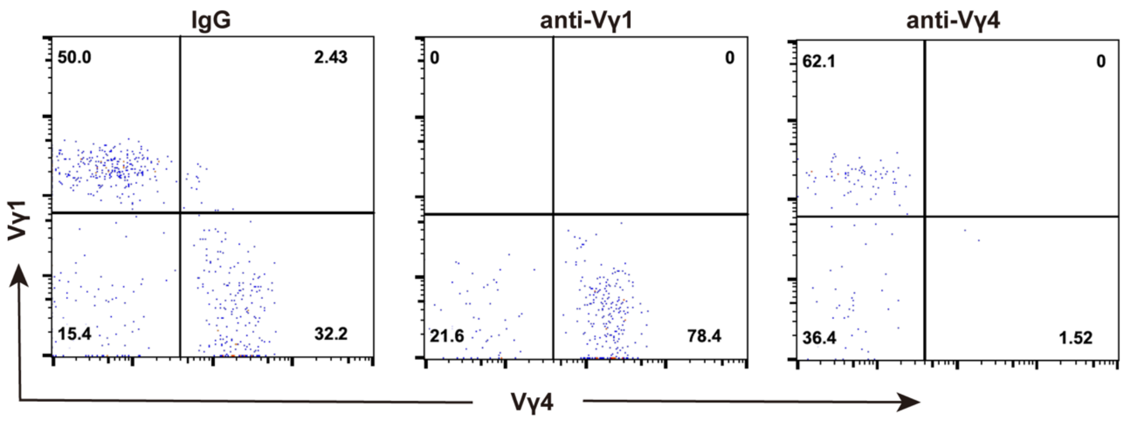

Supplemental Figure 2

Supplemental Figure 2. The depletion of Vγ1 or Vγ4 cells *in vivo*. WT mice were treated with IgG, anti-mouse-Vγ1 or anti-mouse Vγ4 antibody (100ug/200ul/mouse, *iv*). After 1 week, the percentages of Vγ1 and Vγ4 cells in the blood were measured by flow cytometry. Data shown are the representative of two independent experiments.

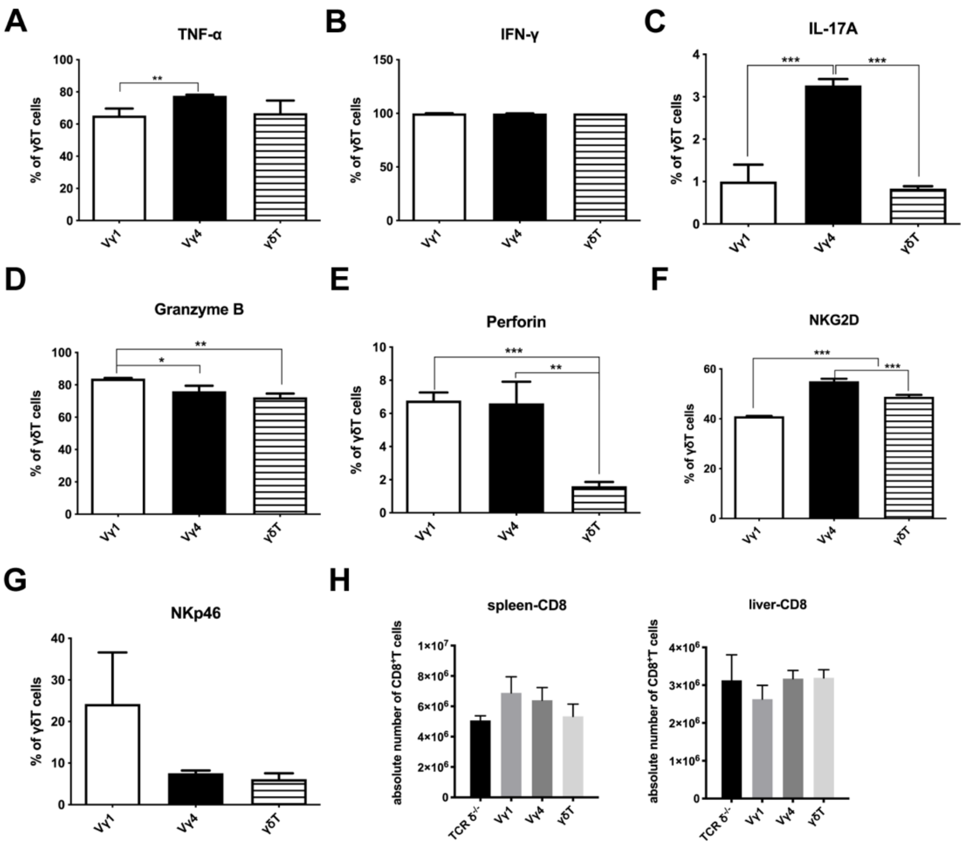

Supplemental Figure 3

Supplemental Figure 3. The phenotypes of expanded Vγ1, Vγ4 and γδT cells *in vitro*. Vγ1, Vγ4 and γδT cells were cultured from the spleen TCR-$\beta^{-/-}$ mouse. The productions of TNFα (A), IFN-γ (B), IL-17A (C), granzyme B (D) and perforin (E) were detected by intracellular staining. The expressions of NKG2D (F) and NKp46 (G) were also examined by flow cytometry (n=3). (H) The absolute number of $CD8^{+}$T cells in the spleen and liver form the recipient mice. Recipient mice were lethally irradiated and received A20 lymphoma cells (1×10^{6}/mouse, *iv.*) plus BM cells (5×10^{6}/mouse, *iv.*) from TCR$\delta^{-/-}$ mice. Vγ1, Vγ4, or total γδT cells (1×10^{7} cells/mouse, *iv.*) from CD45.1-TCR $\beta^{-/-}$ mouse were adoptively transferred into the recipients on day 0. On day7 post allo-HSCT, the absolute number of $CD8^{+}$T cells from the spleen and liver of recipient mice was measured (n=5). Data shown are the representative of at least three experiments. All graphs displayed mean ± SD. *$p < 0.05$, **$p < 0.01$, ***$p < 0.001$ determined by one-way ANOVA followed by Bonferroni posttest.
